# δ^13^C as a Continuum: Tissue‐Based, Ontogenetic, and Interspecific Variation in Carbon Sourcing of a Photosymbiotic Bivalve (Subfamily Fraginae)

**DOI:** 10.1002/ece3.74241

**Published:** 2026-08-28

**Authors:** Aaron Schmidt, Andy D. Y. Tan, Jingchun Li, Lisa Kirkendale, Ruiqi Li

**Affiliations:** ^1^ Department of Ecology and Evolutionary Biology University of Colorado Boulder Boulder Colorado USA; ^2^ Museum of Natural History University of Colorado Boulder Boulder Colorado USA; ^3^ Collections and Research, Western Australian Museum Welshpool Western Australia Australia; ^4^ Department of Biological Sciences University of Southern California Los Angeles California USA

**Keywords:** Fraginae, nutritional mode, ontogeny, photosymbiosis, stable carbon isotopes, tissue heterogeneity

## Abstract

Photosymbiotic marine invertebrates derive carbon from both symbiont photosynthesis and heterotrophic feeding, yet the relative contribution of each source is often inferred from bulk tissue δ^13^C values without accounting for intertissue and intraspecific variations. Here, we measured δ^13^C in three tissues (mantle, gill, and foot) of the photosymbiotic bivalve *Fragum unedo* (subfamily Fraginae) across a range of body sizes from *Gathaagudu* (Shark Bay), Western Australia, and compiled published δ^13^C data from marine mollusks and cnidarians spanning photosymbiotic, chemosymbiotic, and non‐symbiotic nutritional modes. Within *F. unedo*, δ^13^C differed consistently among tissues: the symbiont‐free foot was enriched by approximately 1.1‰ relative to the mantle and 1.6‰ relative to the gill, which is consistent with bulk mixing between host and symbiont biomass in symbiont‐bearing tissue, and also may be due to post‐photosynthetic fractionation during metabolite translocation from symbiont‐bearing source tissues to heterotrophic sink tissues. δ^13^C values also increased with body size, with a total ontogenetic shift of approximately 3‰ between the smallest and largest individuals, suggesting a progressive increase in reliance on symbiont‐derived carbon through growth. Both tissue and size effects are comparable in magnitude to the differences commonly used to distinguish nutritional modes among taxa, indicating that tissue selection and developmental stage can directly alter carbon source assignments. The compiled dataset reveals that chemosymbiotic, photosymbiotic, and non‐symbiotic taxa do not occupy discrete isotopic categories but instead form a continuous δ^13^C gradient with substantial overlap between nutritional modes. The values for *F. unedo* fall within the photosymbiotic range but near its lower boundary, consistent with a mixed nutritional strategy. These results demonstrate that δ^13^C is a valuable tracer of carbon sourcing in symbiotic systems but should be interpreted as a continuous variable shaped by tissue identity, ontogeny, and environmental context rather than as a categorical marker of symbiotic state.

## Graphical abstract

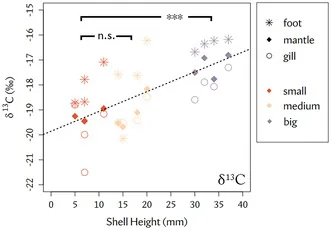

## 1 Introduction

Nutritional symbioses reshape how animals acquire, process, and retain carbon, altering both trophic function and the ecological roles of host lineages in marine ecosystems (Cowen 1988; Stanley and Lipps 2011; Trench 1993). Photosymbioses between marine invertebrates and dinoflagellates are especially important in oligotrophic habitats (Adams et al. 2020), where symbiont‐derived photosynthate can supplement host metabolism and growth in addition to heterotrophic nutrition (Fitt and Trench 1981; Hawkins and Klumpp 1995; J. Li et al. 2018; Ferrier‐Pagès and Leal 2018). These associations, therefore, do not represent a single nutritional mode. Instead, photosymbiotic hosts can vary in the relative contributions of autotrophic carbon fixation by symbionts, inherent heterotrophic feeding by the host, and recycling of nutrients within the holobiont (Guibert et al. 2026). Quantifying how much carbon the host derives from each pathway is therefore essential for understanding the function and dynamics of these symbioses.

Stable carbon isotopes provide a practical way to do this. Animal tissue δ^13^C values reflect the carbon assimilated into biomass, allowing stable carbon isotope measurements to distinguish between heterotrophic food sources and symbiont‐derived carbon (DeNiro and Epstein 1978). In marine consumers, δ^13^C is widely used to distinguish basal carbon sources and overall nutritional modes (Ferrier‐Pagès and Leal 2018; MacKenzie et al. 2011). In symbiotic animals, host δ^13^C varies with the dominant carbon source, whether from photosymbiont fixation, chemosymbiont fixation, or external heterotrophic input. Accordingly, photosymbiotic hosts with Symbiodiniaceae symbionts are often less depleted in ^13^C than comparable non‐symbiotic filter feeders, whereas chemosymbiotic taxa can be more depleted when biomass is largely supported by chemoautotrophic carbon (Rau and Hedges 1979; Robinson and Cavanaugh 1995; Li et al. 2018).

However, δ^13^C cannot be treated as a simple indicator of symbiosis. Isotope values vary among tissues, species, and environmental conditions that influence the balance between photosynthesis and heterotrophic feeding (Guibert et al. 2026; Li et al. 2018). As a result, bulk δ^13^C reflects not only symbiont‐derived carbon but also tissue‐specific metabolism and ecological context. An important unresolved question is how much carbon‐isotope variation exists within a single photosymbiotic species, particularly across tissues and ontogenetic stages. Photosymbiotic cardiid bivalves provide a strong system to address this question because it includes both symbiotic and non‐symbiotic relatives, and symbiotic taxa vary in morphology, habitat, and apparent reliance on symbiont‐derived carbon (Vermeij 2013; Li et al. 2018). In addition, their symbionts are housed in specialized host tissues, including the mantle, gill, and in some species, the foot (Li et al. 2020, 2024), allowing direct comparison of tissues that differ in symbiont abundance and function. Within the subfamily Fraginae, the widespread Indo‐Pacific species *Fragum unedo* inhabits shallow sand habitats and exposes an expanded mantle that enhances light capture for photosymbionts, likely increasing contrasts between symbiont‐rich and symbiont‐poor tissues (ter Poorten 2024). In addition, the availability of multiple individuals spanning an ontogenetic series from *Gathaagudu* (Shark Bay), Western Australia, provides an opportunity to quantify tissue‐ and size‐related isotope variation among individuals occupying similar microenvironments.

Moreover, a broader question is how reliably δ^13^C distinguishes nutritional symbiosis across taxonomic and ecological contexts. Although photosymbiotic, chemosymbiotic, and non‐symbiotic invertebrates are expected to differ in carbon‐isotope values, overlap can arise from mixed nutritional strategies, variation in baseline carbon sources, and differences in tissue sampling (MacKenzie et al. 2011). This limits the use of δ^13^C as a diagnostic tool without comparative context. More comprehensive datasets are needed to define the range of δ^13^C values associated with different nutritional modes and to assess whether values from a focal species align with photosymbiotic taxa, overlap with non‐symbiotic relatives, or reflect within‐species variation linked to tissue type and ontogeny.

Here, we use tissue δ^13^C data from *F. unedo* to test whether reliance on symbiont‐derived carbon changes across ontogeny and whether it differs among host tissues. We measured δ^13^C in symbiont‐rich mantle and gill tissues and symbiont‐free foot tissue across individuals spanning juvenile to adult sizes and evaluated whether variation is best explained by continuous growth, size, tissue type, or their interaction. In addition, we compiled published tissue‐level δ^13^C data from cardiid bivalves and from marine mollusks and cnidarians representing photosymbiotic, chemosymbiotic, and non‐symbiotic lifestyles, providing a comparative framework for interpreting carbon‐isotope values across nutritional modes. Together, these analyses test whether photosymbiotic carbon use is a fixed species‐level trait or varies with host development and tissue identity, and more broadly, how consistently δ^13^C distinguishes among carbon sources across marine symbioses.

## 2 Methods

### 2.1 Sample Collection, Preparation

Twelve *Fragum unedo* individuals were collected from shallow sandy habitats by SCUBA diving and snorkeling at Whalebone Campsite (26.128936° S, 113.638845° E), *Gathaagudu* (Shark Bay), Western Australia in June 2024. Specimens were kept alive overnight, then exposed to sunlight for approximately 7 h, and dissected into symbiont‐rich mantle and gill, and symbiont‐free foot tissues. Tissue samples were dried on foil for 24 h at 60°C and preserved in the refrigerator. Shells and remaining tissues were deposited and vouchered in the Western Australian Museum (WAM) Aquatic Zoology Collection. Specimens were initially classified into small, medium, and large size categories in the field. Specimen shell photos were later taken at WAM, and shell height was measured using ImageJ as a proxy for shell size. Shell heights ranged from 5 to 37 mm, representing juveniles to adults (Data S1). The collection was conducted under DPIRD Fisheries Exemption 250966222 and DBCA Fauna Taking License Reg. 25 FO25000006‐27. Local representatives were contacted during fieldwork in Gathaagudu (Shark Bay), and Malgana Elders, including Senior Ranger Denise Mitchell, were consulted before fieldwork commenced.

### 2.2 Isotope Measurements

Samples were submitted to the Colorado State University EcoCore Analytical Facility for isotope measurements. Sample weighing, acidification, and encapsulation were performed in‐house by them following their standard sample preparation protocol. Stable carbon isotopes were measured using the Delta V IRMS isotope ratio mass spectrometer. Daily offset corrections were applied to account for instrumental drift using glycine standards. δ^13^C values were calculated as (*R*_sample_/*R*_std_ − 1) × 1000 where *R*_sample_ = ^13^C_sample_/^12^C_sample_ and *R*_std_ refer to the Vienna Pee Dee Belemnite Standard (Data S2).

### 2.3 Statistical Analyses

To evaluate the biological predictors of *F. unedo* δ^13^C values, a series of linear and linear mixed‐effects models were constructed. These models evaluated continuous shell height, shell size category (small, medium, large), and tissue type (foot, mantle, gill) as a fixed effect, respectively, alongside a model using shell height as a fixed effect and tissue type as a random effect. The model structures are outlined in Table 1.

**TABLE 1:** Model selection comparison table for linear models (model A–C) and a linear mixed‐effect model (model D) predicting *Fragum unedo* δ^13^C, with marginal (*R* ^2^m) and conditional (*R* ^2^c) variance explained, and Akaike information criterion (AIC) used for model assessment.

Model	Model structure (formula)	Description	*R* ^2^m	*R* ^2^c	AIC
A	δ^13^C ~ height	Only height as fixed effect	0.4539	—	103.40
B	δ^13^C ~ size	Categorical size as fixed effect	0.3985	—	108.50
C	δ^13^C ~ tissue	Only tissue type as fixed effect	0.2569	—	116.41
D	δ^13^C ~ height + (1|tissue)	Height as fixed effect, tissue type as a random effect	0.4681	0.7087	90.90

All statistical modeling and analyses were performed in RStudio v2026.01.1 (Posit Team 2026); with R v4.5.2 (R Core Team 2025), using packages lme4 v1.1.37 (Bates et al. 2015), lmtest v0.9.40 (Zeileis and Hothorn 2002), and MuMIn (Bartoń 2010), for all linear mixed‐effect modeling. Residuals for δ^13^C were verified for normality and homoscedasticity through visual inspection of *Q–Q* plots and residuals versus fitted plots, meeting all assumptions for linear regressions. A Shapiro–Wilk test confirmed that residuals did not significantly deviate from a normal distribution (*W* = 0.9833, *p* = 0.851), and a Breusch–Pagan test indicated no significant heteroscedasticity (*χ* ^2^ = 1.3876, df = 1, *p* = 0.2388). Combinations of fixed and mixed effects were included for model comparisons using primarily Akaike's information criterion (AIC) (Akaike 1974). Marginal *R*‐squared (*R* ^2^m) and conditional *R*‐squared (*R* ^2^c) are reported where applicable. We considered correlations significant at *p* < 0.05.

### 2.4 Comparative Carbon Isotope Data Collection

Scientific literature containing δ^13^C values of bivalves and cnidarians was identified by keyword search on Google Scholar, excluding measurements on bivalve shells and coral skeletons. Sample information such as life stage, nutritional category, tissue type, mean δ^13^C, standard deviation, and sample size were recorded when available. The final comparison dataset contained 126 records from 27 papers (Data S3). To compare carbon isotope across from different phyla and nutrient strategies, records from Mollusca and Cnidaria were grouped by phyla and nutrient strategies (Photosymbiotic, Chemosymbiotic, Non‐symbiotic) and plotted using ggplot2 (Wickham 2009) in R. To further compare the carbon isotope dynamics within the focal group, records from 12 species in the family Cardiidae were grouped and plotted using ggplot2.

## 3 Results

### 3.1 Tissue/Ontogeny Comparison

**FIGURE 1:**
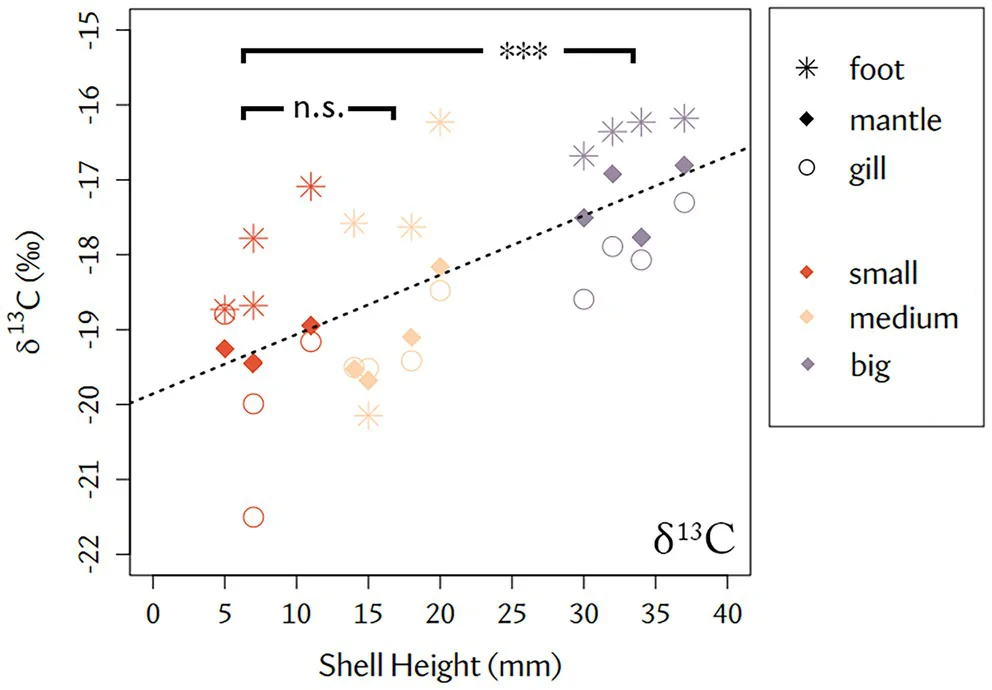
Relationship between *Fragum unedo* shell height (mm) and tissue δ^13^C. Points are color‐coded by shell size categories (small, medium, big) with symbol shapes representing the types of tissues (foot, mantle, or gill). The dashed line represents the linear regression fit (*y* = 0.08*x* − 19.86; *R* ^2^ = 0.46). n.s., not significant; ***, *p* < 0.001.

The 36 *F. unedo* tissue measurements ranged from −21.5‰ to −16.2‰. We found a significant positive relationship (Figure 1) between shell height and δ^13^C (*F*_1,34_ = 29.09, *p* < 0.0001, *R* ^2^ = 0.46), with an increase of δ^13^C by 0.08‰ ± 0.01‰ (mean ± SE) per millimeter of growth.

Model selection identified Model D as the best supported model (Tables 1, 2, 3), which included continuous shell height as a fixed effect and tissue type as a random effect. With shell height as the sole predictor (Model A), 45.4% of the variation in δ^13^C was explained. The inclusion of tissue type as a random intercept (Model D) substantially improved model fit, explaining 70.9% of total variance and indicating significant isotopic signature differences between the tissue types. Specifically, foot tissue (symbiont‐free) was significantly enriched in ^13^C compared to the symbiont‐containing mantle (*p* = 0.0235) and gill tissues (*p* = 0.0018). Our models also showed that shell height captures the size effect sufficiently without needing the categorical binning of size.

**TABLE 2:** Fixed effect term coefficients, standard errors (SE), and accompanying statistics for the linear models.

Term	Estimate	SE	*t*	*p*
Model A: δ^13^C ~ height				
(Intercept)	−19.8568	0.3244	−61.22	**< 0.0001**
Height (mm)	0.07932	0.0147	5.394	**< 0.0001**
Model B: δ^13^C ~ size				
(Intercept)	−19.0686	0.2946	−64.72	**< 0.0001**
Size: medium	0.3208	0.4166	0.7699	0.4468
Size: big	1.8756	0.4166	4.5015	**< 0.0001**
Model C: δ^13^C ~ tissue				
(Intercept)	−17.4429	0.3288	−53.04	**< 0.0001**
Tissue: mantle	−1.1050	0.4651	−2.376	**0.02346**
Tissue: gill	−1.5757	0.4651	−3.388	**0.00184**
Model D: δ^13^C ~ height + (1|tissue)				
(Intercept)	−19.8568	0.4318	−45.98	**< 0.0001**
Height (mm)	0.07932	0.01058	7.499	**< 0.0001**
Random	0.6294	—	—	—
Residual	0.6927	—	—	—

*Note:* Bold text indicates significance at alpha = 0.05.

**TABLE 3:** Analysis of variance (ANOVA) table detailing the fixed effect results for the linear models.

Source of variation	N. DF	D. DF	SS	MS	*F*	*p*	*σ* ^2^
*Model A*							
Height (mm)	1	34	26.986	26.986	29.095	**< 0.0001**	
*Model B*							
Size	2	33	24.152	12.076	11.593	**< 0.001**	
*Model C*							
Tissue	2	33	15.701	7.851	6.050	**0.0058**	
*Model D*							
Height (mm)	1	33	26.987	26.987	56.238	**< 0.0001**	
RE: tissue intercept							0.3962

*Note:* The table shows the sum of squares (SS), degrees of freedom, *F* statistics, and accompanying *p*‐values for each fixed term, alongside the residual variance (*σ* ^2^) in each model. Bold text indicates significance at alpha = 0.05.

### 3.2 Cardiidae Comparison

The focused cardiid comparison contained 20 plotted tissue‐level records from 12 species. The tissue imbalance in this comparison reflects the compiled literature records, which are mostly mantle‐based; in the new *F. unedo* dataset, foot, mantle, and gill were all measured for all individuals. Non‐symbiotic cardiids exhibited consistently depleted δ^13^C values, ranging from −22.4‰ to −19.7‰ (*n* = 4), whereas photosymbiotic cardiids spanned a broader and more enriched range from −19.0‰ to −12.3‰ (*n* = 16).

Within photosymbiotic taxa, δ^13^C values differed among lineages. Giant clams (*Hippopus* and *Tridacna*) generally exhibited less negative δ^13^C values compared to *Fragum* species, although their values overlapped with several *Fragum* taxa. Within the genus *Fragum*, shallow‐water species ( *F. fragum* and *F. whitelyi*) displayed markedly enriched δ^13^C values relative to other congeners. In contrast, *F. unedo*, despite also occurring in shallow habitats, exhibited δ^13^C values similar to *F. sueziense* from deeper environments. Among Tridacninae, δ^13^C values were variable, with *T. derasa* showing the highest values at around −12.7‰ within the group.

### 3.3 Broad Comparison Across Nutritional Modes

The broad metadata comparison contained 105 records from 68 species (Figure 3). Molluscan records spanned all three nutritional categories. Photosymbiotic mollusks ranged from −20‰ to −12‰ (*n* = 27), non‐symbiotic mollusks ranged from about −28‰ to −16‰ (*n* = 46), and chemosymbiotic mollusks ranged from −39‰ to −25‰ (*n* = 18). Non‐symbiotic and photosymbiotic records overlapped by around 4‰ while chemosymbiotic and non‐symbiotic records overlapped by around 3‰. All cnidarian records in the compiled dataset were photosymbiotic and ranged from about −19‰ to −13‰ (*n* = 14).

For Mollusca, δ^13^C values for each nutritional category were largely distinct with small amounts of overlap; chemosymbiotic mollusks were consistently the most depleted, forming a distinct cluster below −30‰, with no overlap with other groups except for one outlier from *Thyasira peregrina*. Non‐symbiotic mollusks occupied an intermediate range, overlapping at the upper end with photosymbiotic mollusks that are generally more enriched in ^13^C. Comparison between photosymbiotic mollusks and cnidarians revealed substantial overlap in δ^13^C values, particularly between −18‰ and −14‰.

## 4 Discussion

### 4.1 Post‐Photosynthetic Fractionation May Contribute to Tissue‐Specific δ^13^C Variations

Consistent differences in δ^13^C were observed among tissues in *Fragum unedo*, with the foot enriched relative to the mantle and gill, and the mantle slightly enriched relative to the gill (foot–mantle about 1.1‰, foot–gill about 1.6‰, mantle–gill about 0.5‰). The same pattern was reported in the other photosymbiotic *Fragum* species, where foot tissue is enriched by approximately 0.5‰–1.5‰ relative to symbiont‐bearing mantles (Li et al. 2018). Since this pattern recurs independently across species, locations, and ontogenetic stages, it is unlikely to be coincidental and instead indicates that tissue identity contributes systematically to within individual δ^13^C variation in photosymbiotic fragines.

These differences may have arisen in part from the presence of symbionts in mantle and gill tissues, which would lower bulk δ^13^C through mixing of host and symbiont carbon. In photosymbiotic giant clams, host tissues are typically enriched in ^13^C relative to symbionts by about 0.5‰–1.7‰ (Guibert et al. 2026). However, the larger difference observed here, particularly between foot and gill, suggests that symbiont biomass mixing alone cannot explain the full magnitude of tissue‐level variation. Post‐photosynthetic fractionation may offer a complementary explanation. In terrestrial plants, autotrophic organs (leaves) are consistently 1‰–2‰ more ^13^C‐depleted than heterotrophic tissues such as roots and woody stems (Badeck et al. 2005). Two processes may drive this pattern: First, preferential respiratory release of ^13^C‐enriched CO_2_ from source organs leaves behind organic matter that is relatively ^13^C‐depleted (Badeck et al. 2005). Second, export of ^13^C‐enriched metabolites (e.g., sucrose) from source to sink organs enriches the heterotrophic sink tissues. An analogous mechanism may operate in *F. unedo*: ^13^C‐enriched metabolites translocated from symbiont‐bearing mantles and gills to the foot would enrich the foot relative to its source tissues, while respiratory ^13^C loss from the metabolically active mantle and gill would further deplete those organs (Badeck et al. 2005). Differences in biochemical composition may further contribute, since lipids are ^13^C depleted relative to proteins and carbohydrates, and higher lipid content in mantle could shift their δ^13^C to lower values (McConnaughey and McRoy 1979; Post et al. 2007). Compound‐specific isotope analysis of fatty acids in the *Tridacna gigas* symbiosis provides direct evidence that specific metabolites are translocated with minimal fractionation: palmitic acid and palmitoleic acid had matching δ^13^C values in zooxanthellae and all host tissues, whereas 18‐carbon acids in host tissues were significantly ^13^C‐depleted relative to zooxanthellae, indicating derivation from heterotrophic feeding rather than from the photosymbionts (Johnston et al. 1995). This selective translocation of certain lipid species shows that the isotopic enrichment of sink tissues depends not only on the bulk δ^13^C of translocated carbon but also on the metabolite composition of the exported pool.

**FIGURE 2:**
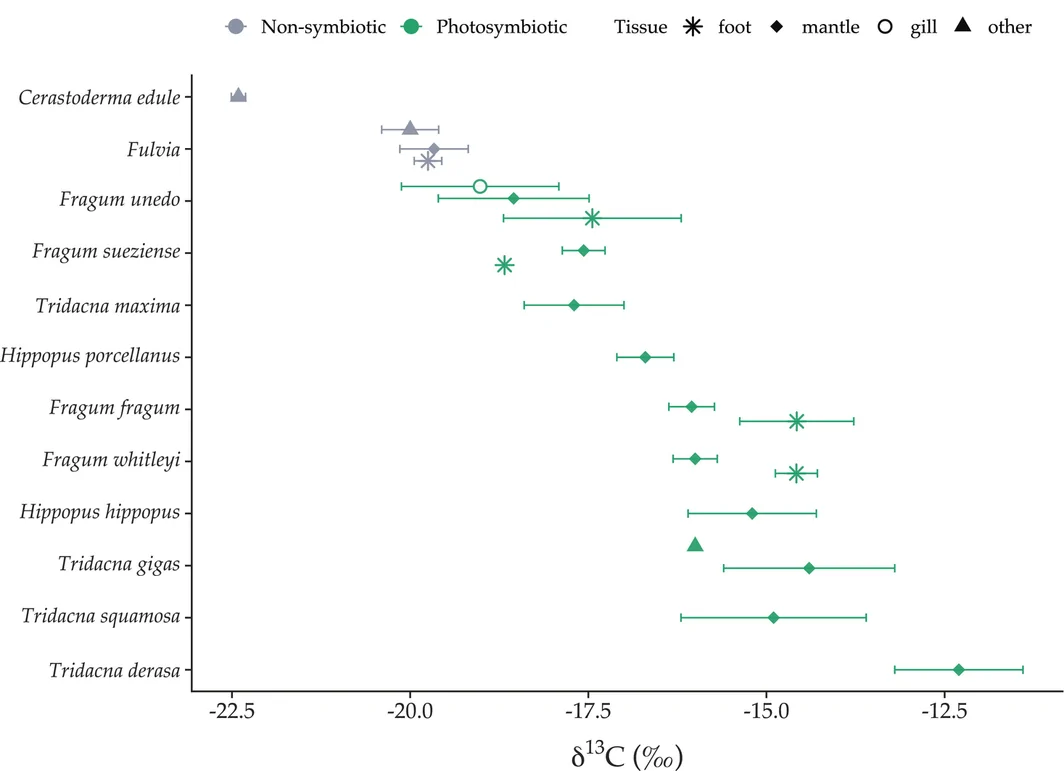
Focused comparison of δ^13^C values in cardiid bivalves. Points represent tissue‐level mean δ^13^C, ordered by taxon‐level mean δ^13^C. Colors indicate nutritional category and shapes indicate tissue group. Horizontal bars show standard deviations (SD) when reported in the original source; no bar is shown when SD was not available. Sample sizes for each record are provided in Data S3.

The within‐individual variation observed here, approaching 2‰, is comparable to the differences commonly used to distinguish nutritional modes or carbon sources among taxa. For instance, the offset between photosymbiotic *F. unedo* and non‐symbiotic *Fulvia* falls within this same range (Figure 2). Therefore, tissue selection directly affects inferred carbon source assignments, and δ^13^C values from a single tissue should not be assumed to represent whole‐organism isotopic composition. In addition, the inter‐tissue variation reflects a key limitation of the present study: bulk measurements combine host and symbiont biomass, making it impossible to partition host cellular δ^13^C from symbiont δ^13^C in mantle and gill samples. Future work should physically separate host and symbiont fractions (e.g., through differential centrifugation or cell sorting) to determine how much of the observed tissue‐level offset is attributable to symbiont biomass mixing versus host metabolic fractionation. Compound‐specific isotope analysis of metabolites would further identify which carbon pools are transferred between symbionts and host tissues and where fractionation occurs along this pathway. Identifying the specific metabolites exported from symbiont‐bearing tissues, whether as glucose, glycerol, or amino acids, would help constrain the isotopic enrichment expected in sink tissues and allow quantitative modeling of post‐photosynthetic fractionation in marine photosymbioses.

### 4.2 Ontogenetic Shift in δ^13^C and Increasing Photosymbiont Reliance

δ^13^C values increased significantly with shell height, with larger individuals exhibiting less negative values. The total ontogenetic shift approaches ~3‰ between the smallest and largest individuals, comparable to differences used to distinguish carbon sources among taxa (Figure 1). As with inter‐tissue variations, this magnitude means that specimen selection across ontogenetic stages can alter inferred carbon source assignments. Similarly, the scale of this shift overlaps with the separation between symbiotic and non‐symbiotic taxa, so developmental stage alone can change the nutritional mode reflected by carbon isotopes. Size and developmental stages should therefore be documented and accounted for in isotopic comparisons.

In photosymbiotic systems, less negative δ^13^C values are generally associated with a greater contribution of symbiont‐fixed carbon relative to heterotrophic inputs (Ferrier‐Pagès and Leal 2018; Li et al. 2018). The positive relationship between size and δ^13^C, therefore, suggests an increasing reliance on symbiont‐derived carbon through ontogeny. This pattern is consistent with previous studies in giant clams. Killam et al. (2023) showed that δ^15^N declines through shell growth in *Tridacna*, reflecting a shift from heterotrophy to symbiont dependence, and Yau and Fan (2011) reported increased photosynthetic performance with size. Our observation in *F. unedo* follows the same directional trend documented in giant clams, which may reflect a general pattern in photosymbiotic cardiids.

Several mechanisms could generate this ontogenetic trend. As body size increases, surface area to volume ratios decline, which may reduce the efficiency of suspension feeding relative to metabolic demand (Hawkins and Klumpp 1995). At the same time, the expansion of symbiont‐bearing tissues could increase total photosynthetic capacity. In giant clams, symbiont density scales with body mass through the development of extensive internal tissue systems (Holt et al. 2014). A comparable process may occur in *F. unedo*, where leaf‐like mantle extensions that host dense symbiont populations could become more developed with growth, increasing the effective area for light capture. Unlike taxa with specialized shell modifications such as windows and flattened shells of *Corculum* (ter Poorten 2024), *F. unedo* may rely primarily on mantle expansion to support photosymbiosis, suggesting an adaptation strategy convergent with giant clams.

However, this shift may not be driven solely by increased symbiont capacity. As in other fragines, *F. unedo* harbors symbionts within the gills (Kirkendale 2009). Symbiont colonization of the gills could progressively compromise the ciliary and mucus‐based mechanisms used for particle capture and sorting, reducing filter‐feeding efficiency in larger individuals and further tilting the carbon budget toward photosymbiont reliance. The carbon budget would shift toward symbiont‐derived inputs even without a proportional increase in symbiont productivity. The ontogenetic increase in δ^13^C therefore likely reflects both elevated autotrophic input and reduced heterotrophic intake, rather than a single underlying process. Direct measurements of feeding rates, symbiont biomass, and tissue‐specific carbon fluxes are needed to determine the relative contributions of these processes and confirm the mechanisms underlying the ontogenetic δ^13^C shift.

### 4.3 δ^13^C as a Continuum: From Fixation Biochemistry to Ecological Inference

**FIGURE 3:**
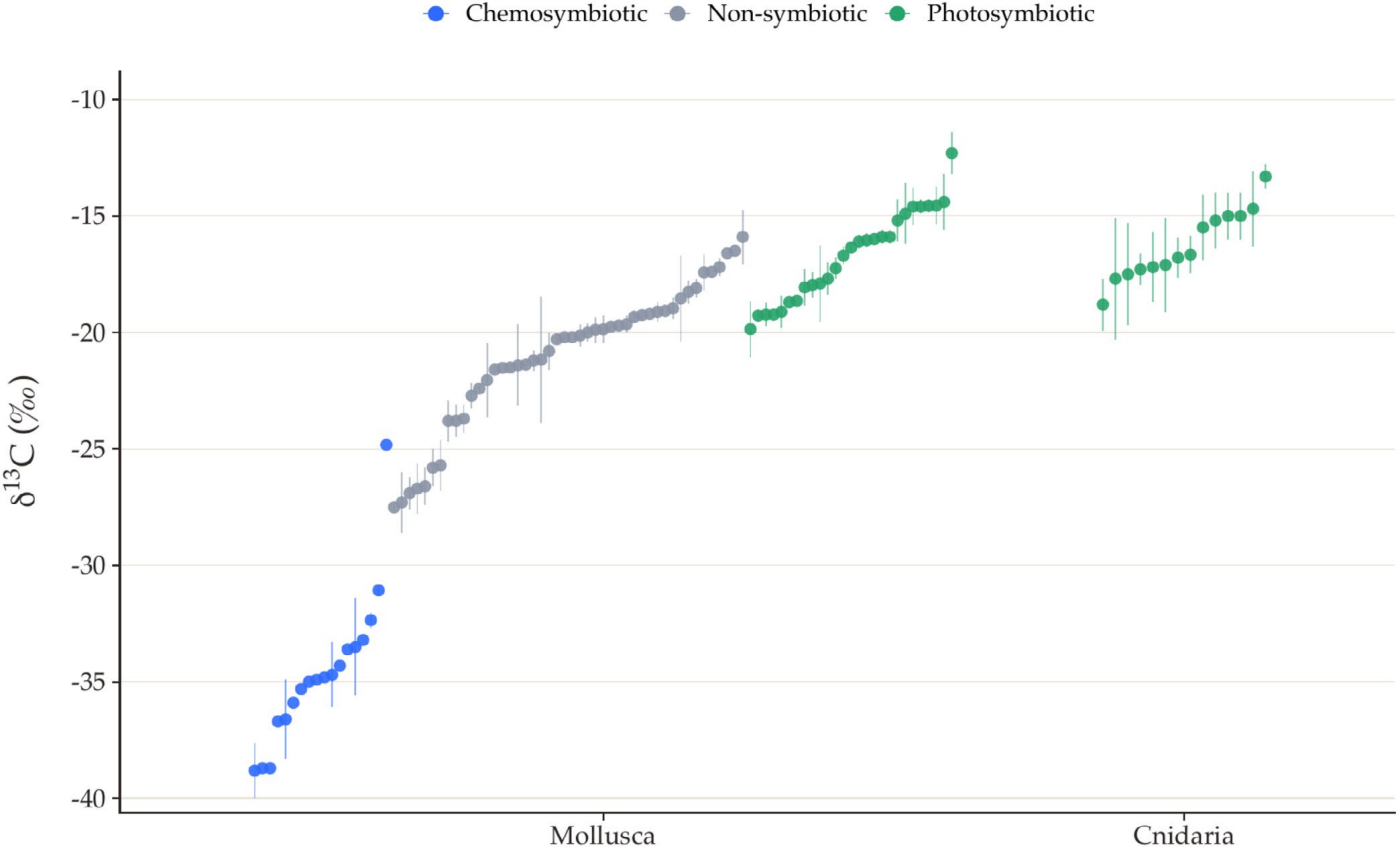
Broad comparison of δ^13^C values in fresh tissues across molluscan and cnidarian records, including compiled literature values and the new data generated in this study. Points represent means grouped by phylum, with error bars showing standard deviations when reported. Colors indicate chemosymbiotic (blue), non‐symbiotic (gray), and photosymbiotic (green) lifestyles. Source data are provided in Data S3.

Bulk tissue δ^13^C reflects the isotopic composition of assimilated carbon with only minor trophic enrichment (DeNiro and Epstein 1978). In symbiotic systems, the relative contributions of autotrophic and heterotrophic carbon sources determine where an organism falls along the δ^13^C spectrum. The compiled data (Figure 3) show that chemosymbiotic, non‐symbiotic, and photosymbiotic taxa do not form discrete isotopic categories but instead occupy a continuous gradient, chemosymbiotic mollusks at the most ^13^C‐depleted end, photosymbiotic taxa at the most ^13^C‐enriched end, and non‐symbiotic filter feeders distributed across intermediate values. Each nutritional mode draws carbon from sources with distinct isotopic baselines, but the contribution of each source varies continuously among taxa and within individuals, producing broad and partially overlapping ranges.

The strongly depleted values of chemosymbiotic mollusks (below −30‰ in most cases) arise from the isotopic signatures of chemoautotrophic carbon fixation. Thioautotrophic symbionts using form II RuBisCO in the Calvin–Benson–Bassham cycle fractionate carbon more strongly than form I, producing biomass that is markedly ^13^C‐depleted relative to dissolved inorganic carbon (Robinson and Cavanaugh 1995; Scott et al. 2004). Photosymbiotic taxa occupy the opposite end of the gradient. Symbiodiniaceae fix carbon using form I RuBisCO under high light and often CO_2_‐limited conditions, which reduces discrimination against ^13^C and produces less negative δ^13^C values in host tissues. In giant clams and corals, carbon limitation at the within host site of fixation further enriches the photosynthate, yielding values as high as −10‰ to −12‰ (Muscatine et al. 1989). In addition, non‐symbiotic filter feeders span the middle of the gradient. Their δ^13^C values track the isotopic composition of local particulate organic matter, which integrates contributions from phytoplankton, detrital material, and dissolved organic carbon (Burkhardt et al. 1999).

Although fixation biochemistry sets the approximate range for each nutritional mode, several factors shift values within those ranges. Photosynthetic discrimination against ^13^C varies with the balance between carbon supply and demand at the site of fixation (Farquhar et al. 1989). When supply exceeds demand, RuBisCO more fully expresses its kinetic preference for ^12^C and biomass becomes more depleted in δ^13^C; when demand approaches supply, discrimination decreases and biomass becomes less negative in δ^13^C. Light, nutrients, growth rate, temperature, and dissolved inorganic carbon availability all alter this balance. In marine phytoplankton alone, δ^13^C ranges from approximately −18‰ to −24‰ depending on cell size, growth rate, and CO_2_ concentration (Burkhardt et al. 1999). Seagrasses under carbon limitation can reach −7‰ to −8‰ (Duarte et al. 2018; Hemminga and Mateo 1996), while phytoplankton in the same region may be 15‰ more depleted. Consumer tissues inherit this producer‐side variation, so two filter feeders at identical trophic levels can differ substantially in δ^13^C simply because they fed on production with different isotopic baselines (MacKenzie et al. 2011). In symbiotic systems, the host‐symbiont δ^13^C offset itself changes seasonally (Swart et al. 2005), and species within the same photosymbiotic clade vary in degree of autotrophy versus heterotrophy along a continuum rather than occupying a single isotopic position (Guibert et al. 2026).

The spread within each category, therefore, represents real ecological variation. In photosymbiotic systems, differences in symbiont abundance, light exposure, and internal recycling can shift δ^13^C, as can tissue‐specific metabolism and ontogeny, consistent with the patterns observed in *F. unedo* (Omata et al. 2008; Treignier et al. 2009). Variation in non‐symbiotic taxa reflects differences in diet and baseline carbon sources, while chemosymbiotic variability arises from differences in fixation biochemistry and environmental carbon supply (Dreier et al. 2012; Guibert et al. 2026). The overlap among ranges of different nutritional modes in our dataset follows directly from these processes. As a result, δ^13^C is most informative when interpreted in context. The values for *F. unedo* are consistent with photosymbiosis, but their position at the lower end of the range, together with clear tissue and size effects, shows that δ^13^C should be treated as a continuous indicator of carbon sourcing rather than a fixed diagnostic of nutritional mode.

This variability requires caution when using δ^13^C to infer nutritional mode, particularly in fossil systems where environmental context is poorly constrained. Fossil symbiosis inferences have often relied on shell or skeletal carbonate δ^13^C when symbionts themselves are not preserved, such as in corals (Stanley and Swart 1995; Frankowiak et al. 2016) and foraminiferans (Houston and Huber 1998). However, shell and skeletal carbonate δ^13^C are not direct metabolic indicators. In addition, without independent constraints on baseline isotopic composition, water depth, and light regime, δ^13^C alone cannot reliably distinguish these scenarios, as illustrated by conflicting interpretations of photosymbiosis in Mesozoic reef bivalves (Lipps and Stanley 2016). Pairing δ^13^C with complementary proxies, such as shell‐bound δ^15^N sclerochronology (Killam et al. 2023) or compound‐specific metabolites isotope analysis (Ferrier‐Pagès and Leal 2018), offers a more robust path toward identifying photosymbiosis in both living and fossil taxa. The δ^13^C gradient documented here provides a useful comparative framework, but individual values should not be treated as categorical markers of symbiotic state without accounting for the environmental and biological factors that shape them.

## 5 Conclusions

Our results show that δ^13^C in the photosymbiotic bivalve *Fragum unedo* varies systematically with both tissue type and body size, demonstrating that reliance on symbiont‐derived carbon is not a fixed species‐level trait but shifts with host development and differs among organs. Tissue‐specific offsets approaching 2‰ between symbiont‐rich and symbiont‐free tissues point to post‐photosynthetic fractionation and symbiont biomass mixing as sources of within‐individual isotopic variation, while the ~3‰ ontogenetic shift toward less negative δ^13^C in larger individuals indicates a progressive increase in autotrophic carbon use, paralleling patterns documented in giant clams. Both sources of variation are large enough to alter inferred carbon source assignments, and their magnitudes overlap with the differences commonly used to distinguish nutritional modes among taxa. The compiled δ^13^C metadata further confirms that chemosymbiotic, photosymbiotic, and non‐symbiotic mollusks and cnidarians form a continuous δ^13^C gradient rather than discrete isotopic categories, with substantial overlap between adjacent nutritional modes. δ^13^C therefore remains a valuable tracer of carbon sourcing in symbiotic systems, but its interpretation requires attention to tissue type, ontogenetic stage, and environmental context. Future work separating host from symbiont carbon fractions and applying compound‐specific isotope approaches will be essential for resolving the metabolic pathways that underlie these patterns and for strengthening δ^13^C‐based inference of photosymbiosis in both living and fossil taxa.

## Author Contributions

**Aaron Schmidt:** formal analysis (equal), investigation (equal), writing – original draft (equal). **Andy D. Y. Tan:** conceptualization (equal), formal analysis (equal), writing – review and editing (equal). **Jingchun Li:** conceptualization (equal), funding acquisition (equal), resources (equal), writing – review and editing (equal). **Lisa Kirkendale:** conceptualization (equal), funding acquisition (equal), resources (equal), writing – review and editing (equal). **Ruiqi Li:** conceptualization (equal), formal analysis (equal), investigation (equal), supervision (equal), visualization (equal), writing – original draft (lead), writing – review and editing (lead).

## Funding

This work was supported by Packard Fellowship for Science and Engineering (2019711‐69653).

## Conflicts of Interest

The authors declare no conflicts of interest.

## Supporting information


**Data S1:** Specimen metadata for *Fragum undeo* used in this study.
**Data S2:** Stable carbon isotope and shell height measurements for *Fragum unedo*.
**Data S3:** Compiled literature dataset.

## Data Availability

Raw isotope data and comparative metadata are available as Data S1–S3. All analysis scripts, including the model selection and plotting code, are openly accessible at the following GitHub repository (https://github.com/Ruiqi‐CUB/2026_FragumIsotope).
